# Vitamin D deficiency causes airway hyperresponsiveness, increases airway smooth muscle mass, and reduces TGF‐*β* expression in the lungs of female BALB/c mice

**DOI:** 10.1002/phy2.276

**Published:** 2014-03-26

**Authors:** Rachel E. Foong, Nicole C. Shaw, Luke J. Berry, Prue H. Hart, Shelley Gorman, Graeme R. Zosky

**Affiliations:** 1 Telethon Institute for Child Health Research, The University of Western Australia, Subiaco, Western Australia, Australia; 2 School of Medicine, Faculty of Health, University of Tasmania, Hobart, Tasmania, Australia

**Keywords:** Airway hyperresponsiveness, airway smooth muscle, lung structure, mouse model, vitamin D

## Abstract

Vitamin D deficiency is associated with disease severity in asthma. We tested whether there is a causal association between vitamin D deficiency, airway smooth muscle (ASM) mass, and the development of airway hyperresponsiveness (AHR). A physiologically relevant mouse model of vitamin D deficiency was developed by raising BALB/c mice on vitamin D‐deficient or ‐replete diets. AHR was assessed by measuring lung function responses to increasing doses of inhaled methacholine. Five‐micron sections from formalin‐fixed lungs were used for ASM measurement and assessment of lung structure using stereological methods. Transforming growth factor (TGF)‐*β* levels were measured in bronchoalveolar lavage fluid (BALF). Lungs were dissected from embryonic day (E) 17.5 vitamin D‐deficient and ‐replete fetal mice for quantification of ASM density and relative gene expression of TGF‐*β* signaling pathway molecules. Eight‐week‐old adult vitamin D‐deficient female mice had significantly increased airway resistance and ASM in the large airways compared with controls. Vitamin D‐deficient female mice had a smaller lung volume, volume of parenchyma, and alveolar septa. Both vitamin D‐deficient male and female mice had reduced TGF‐*β* levels in BALF. Vitamin D deficiency did not have an effect on ASM density in E17.5 mice, however, expression of TGF‐*β*1 and TGF‐*β* receptor I was downregulated in vitamin D‐deficient female fetal mice. Decreased expression of TGF‐*β*1 and TGF‐*β* receptor I during early lung development in vitamin D‐deficient mice may contribute to airway remodeling and AHR in vitamin D‐deficient adult female mice. This study provides a link between vitamin D deficiency and respiratory symptoms in chronic lung disease.

## Abstract (precis)

Vitamin D deficiency caused airway hyperresponsiveness and increased airway smooth muscle mass in the airways of adult female mice. Vitamin D deficiency also reduced transforming growth factor (TGF)‐*β*1 protein levels in both male and female mice, as well as reduced gene expression of TGF‐*β*1 and TGF‐*β* receptor I in female E17.5 fetal pups. These observations may provide a link between vitamin D deficiency and respiratory symptoms in chronic lung disease.

## Introduction

Vitamin D is a steroid hormone that has long been known to play an important role in calcium homeostasis within the body. The recent discovery that the vitamin D receptor (VDR) is ubiquitously expressed in nearly every cell type in human tissue has led to the realization that vitamin D also has extraskeletal effects (Bouillon et al. 2008) and there is now a large body of evidence linking vitamin D deficiency to chronic conditions, including autoimmune, infectious, cardiovascular, and respiratory disease (Munger et al. 2006; Holick 2007; Ginde et al. 2009; Anderson et al. 2010; Chinellato et al. 2011). Of relevance to chronic respiratory disease, some studies have shown that vitamin D deficiency is more prevalent in patients with asthma and chronic obstructive pulmonary disease (COPD), (Janssens et al. 2010; Chinellato et al. 2011) and lower vitamin D levels have been associated with poor asthma control, increased corticosteroid use (Searing et al. 2010), and airway hyperresponsiveness (AHR; Sutherland et al. 2010).

Epidemiological and experimental studies have also shown that lower serum vitamin D levels are associated with reduced lung function (Black and Scragg 2005; Zosky et al. 2011). Recently, Gupta et al. (2011) detected an inverse relationship between vitamin D levels, asthma severity, and inhaled steroid use in children with moderate and severe asthma. Interestingly, lower serum vitamin D levels were also associated with an increase in airway smooth muscle (ASM) mass, measured from endobronchial biopsy samples, in children with severe asthma. This apparent capacity of vitamin D to modulate ASM growth is supported by microarray studies showing that the vitamin D receptor is expressed in ASM cells (Bosse et al. 2007).

1,25‐Dihydroxyvitamin D (1,25(OH)_2_D_3_), the active form of vitamin D, can also inhibit ASM cell proliferation in vitro (Damera et al. 2009) and transforming growth factor (TGF)‐*β*‐induced expression of *α*‐smooth muscle actin in myofibroblasts (Ramirez et al. 2010). TGF‐*β* has profibrotic properties and can induce epithelial–mesenchymal transition, which may mediate airway remodeling (Hackett et al. 2009). Epithelial–mesenchymal interactions also play a crucial role in lung development and can be promoted by vitamin D (Sakurai et al. 2009). While the study by Gupta et al. may provide in vivo evidence that vitamin D levels are associated with ASM remodeling in children with severe asthma, the cross‐sectional nature of the study prevents demonstration of a causal association between vitamin D and ASM (Gupta et al. 2011). Furthermore, vitamin D deficiency is associated with physical inactivity (Strine et al. 2007), which in turn is linked to the severity of asthma (Brock et al. 2010). As such, vitamin D may simply be acting as an indirect marker of physical activity levels.

On the basis of the in vitro evidence and the clinical data provided by Gupta et al. (2011), we hypothesize that vitamin D deficiency alters lung structure and airway structure by increasing ASM mass, leading to AHR, a characteristic feature of asthma. We tested this hypothesis by investigating whether vitamin D‐deficient mice showed evidence of airway remodeling and airway hyperresponsiveness. We also investigated the potential role of TGF‐*β* in modulating the effects we observed.

## Materials and Methods

### Mouse model

Three‐week‐old female BALB/c mice (Animal Resource Centre, Murdoch, WA, Australia) were placed on either vitamin D_3_‐deficient or ‐replete diets containing 0 or 2280 IU of vitamin D_3_, respectively, (Specialty Feeds, Glen Forrest, WA, Australia) as described previously (Gorman et al. 2012b). Deficient diets were supplemented with 2% (vs. 1%) calcium to prevent hypocalcaemia. This level of supplementation is sufficient to maintain serum calcium and prevent bone defects in vitamin D‐deficient mice (Zosky et al. 2011). Mice were housed in a room with a 12‐h ultraviolet B‐free light/dark cycle. At 8 weeks of age, female mice were mated with vitamin D‐replete male BALB/c mice. Offspring of both sexes were maintained on deficient or replete diets and studied at 8 weeks of age.

To obtain fetal tissue samples, females were time‐mated. The morning after the first observation of a vaginal plug was designated as day 0.5 of gestation. On embryonic day (E) 17.5, which corresponds with a period of substantial airway development (canalicular stage of lung development), dams were euthanized by overdose with ketamine (Troy Laboratories, Glendenning, NSW, Australia): xylazine (Troy Laboratories) at a dose of 800 mg/kg: 40 mg/kg, by intraperitoneal injection and pups obtained by cesarean section. Serum 25‐hydroxyvitamin D (25(OH)D_3_) levels were measured using IDS OCTEIA ELISA kits (Immunodiagnostic Systems Ltd, Boston Business Park, UK). All procedures were approved by the Telethon Institute for Child Health Research Animal Ethics Committee and conformed to National Health and Medical Research Council (NHMRC) of Australia guidelines.

### Animal preparation for lung function assessment

Mice were anesthetized by intraperitoneal injection with a solution containing 40 mg/mL of ketamine and 2 mg/mL of xylazine at a dose of 0.01 mL/g body weight. Two‐thirds of the dose was administered initially to induce surgical anesthesia prior to tracheostomy and cannulation with a polyethylene endotracheal tube (internal diameter = 0.086 cm, length = 1.0 cm). Mice were placed in a whole‐body plethysmograph and connected to a small animal ventilator (HSE‐Harvard MiniVent; Harvard Apparatus, Holliston, MA). The remaining anesthetic was given and mice ventilated at 400 breaths/min with a tidal volume of 10 mL/kg and 2 cm H_2_O positive end‐expiratory pressure.

### Lung volume

Thoracic gas volume (TGV) was measured by plethysmography as described previously (Janosi et al. 2006). During periods of apnea, the trachea, and box were occluded at elastic‐equilibrium lung volume (EELV). Intramuscular electrodes were used to stimulate the intercostal muscles and induce inspiratory efforts. TGV was calculated by applying Boyle's Law to the tracheal and box pressure signals after correction for the thermal properties of the plethysmograph (Janosi et al. 2006).

### Methacholine challenge

After performing lung volume measurements, mice were transferred to a flexiVent system (SCIREQ, Montreal, QC, Canada) for assessment of responsiveness to methacholine (*β*‐methacholine chloride, Sigma–Aldrich, St. Louis, MO). Respiratory system input impedance (Zrs) was measured using a modification in the low‐frequency forced oscillation technique (LFOT). Lung volume history was standardized using three slow inflation–deflation maneuvres up to 20 cm H_2_O transrespiratory pressure before measurement of baseline lung mechanics. During pauses in ventilation, a 16‐sec oscillatory signal containing 19 frequencies ranging from 0.25 to 19.625 Hz was delivered by the flexiVent piston in order to measure Zrs. A four‐parameter model with constant phase tissue impedance (Hantos et al. 1992) was then fitted to the data for calculation of the Newtonian resistance (*R*_aw_, which primarily reflects airway resistance in the mouse due to the low chest wall impedance), tissue damping (*G*, the parameter describing the viscous frequency‐dependent response in resistance which is thought to reflect resistance of the peripheral airways where airflow occurs primarily by diffusion) and tissue elastance (*H*, the elastic properties of the lung parenchyma). LFOT measurements were taken once a minute for 5 min to establish baseline lung mechanics. Mice were then challenged (10 sec) with increasing concentrations of aerosolized methacholine from 0.3 mg/mL up to 100 mg/mL. Five LFOT measurements were taken after each dose of methacholine, and the maximum responses for *R*_aw_, *G,* and *H* from these measurements were used to construct dose–response curves and compare responses between groups. The dose of methacholine required to cause a doubling in *R*_aw_ (EC_200_; as a measure of sensitivity to methacholine) was calculated using linear interpolation between log doses of methacholine from the full dose–response curve.

### Lung structure

Lung structure was assessed in separate groups of mice according to ATS/ERS guidelines (Hsia et al. 2010). After euthanasia, lungs were inflation‐fixed with 4% formaldehyde solution at a pressure of 10 cm H_2_O. The left lung was then separated from the right lung at the main bronchus. The left lobe was embedded in paraffin with the transverse section face down to obtain cross‐sections of the first‐generation airway. The right lung was randomly orientated (Nyengaard and Gundersen 2006) and embedded in a separate paraffin block. Starting at a random point, 5‐*μ*m sections were taken at regular 500 *μ*m intervals throughout the right lung and stained with hematoxylin and eosin. Lung structure was assessed in the right lung using stereological methods as described previously (Zosky et al. 2011).

### ASM measurements and immunohistochemistry

The left lung was used for assessment of ASM mass. Five‐micron‐thick transverse sections were cut at 500 *μ*m intervals throughout the entire lung. These sections coincide with the main bronchus which traverses to the base of the left lung (Thiesse et al. 2010) allowing sections from the central to the peripheral airways to be obtained. Large airways consisted of the primary bronchus with a perimeter of basement membrane >2000 *μ*m (mean area and diameter were 517185 *μ*m^2^ and 982 *μ*m, respectively). Small airways were designated as peripheral airways distal to the primary bronchus with a basement membrane perimeter <1000 *μ*m (mean area and diameter were 113281 *μ*m^2^ and 226 *μ*m, respectively). Sections were then stained with Masson's Trichrome for visualization of structural elements. Immunohistochemistry for *α*‐smooth muscle actin (*α*‐SMA [ab5694]; 1:10000 dilution; Abcam, Cambridge, MA) was carried out to confirm the presence of contractile elements. The cross‐sectional area of ASM and internal perimeter of the basement membrane were measured using the newCast stereology software (Visiopharm, Denmark). The square root of ASM area was then corrected by the perimeter of the basement membrane (James et al. 1988).

Due to the small size of the lung samples from E17.5 pups and the difficulty in accurately determining orientation so as to ensure transverse section of the main conducting airways, a stereological approach was used to quantify ASM. Briefly, whole E17.5 lungs were embedded in paraffin and randomly oriented. Immunohistochemistry for *α*‐SMA was carried out on 5‐*μ*m lung sections and the percentage of cells staining positive for *α*‐SMA was calculated using stereological techniques.

### Measurement of TGF‐*β* levels

Bronchoalveolar lavage fluid (BALF) was collected from separate groups of 8‐week‐old mice by slowly washing 0.4 mL of saline in and out of the lungs three times. TGF‐*β* protein levels in the BALF were measured using a DuoSet ELISA Development kit for measuring TGF‐*β*1 (R&D Systems, Minneapolis, MN) following the manufacturer's instructions.

### Quantification of gene expression

RNA was extracted from lung homogenates using an RNeasy^®^ Plus Mini Kit (Qiagen Inc, Valencia, CA) according to the manufacturer's instructions. Concentration and quality of the RNA extract were assessed using a ND‐1000 Nanodrop spectrophotometer (Thermo Scientific, Waltham, MA). Approximately 150 ng of RNA was converted to cDNA by reverse transcriptase PCR using the Quick‐Start Protocol supplied in the Omniscript Reverse Transcriptase kit (Qiagen Inc.). The cDNA was then amplified by real‐time PCR using commercially available TaqMan hydrolysis probes (Applied Biosystems, Foster City, CA) for TGF‐*β*1, TGF‐*β*2, TGF‐*β*3, TGF‐*β* receptor I, TGF‐*β* receptor II and TGF‐*β* receptor III according to the setup specified in the TaqMan gene expression master mix protocol (Applied Biosystems). The amplification reactions were performed using an ABI PRISM 7900HT Sequence Detection System version 3.0 (Applied Biosystems). After adjustment of the baseline and threshold for each detector, threshold cycle (Ct) values were normalized to the reference gene, 18s ribosomal RNA (Applied Biosystems), by a ΔCt calculation (Ct_target gene_ − Ct_reference gene_). All statistics were performed on transformed ΔCt values.

### Statistical analysis

Between group comparisons, within sexes, were made using *t* tests or repeated‐measures analysis of variance (ANOVA). Statistical analyses were performed using SigmaPlot software (version 12 SysStat Software, Chicago, IL). Data were logtransformed where required to satisfy the assumptions of normality for *t* tests and data are shown as mean (SD). *P* values of <0.05 were regarded as statistically significant.

## Results

### Model characteristics

**Figure 1.**
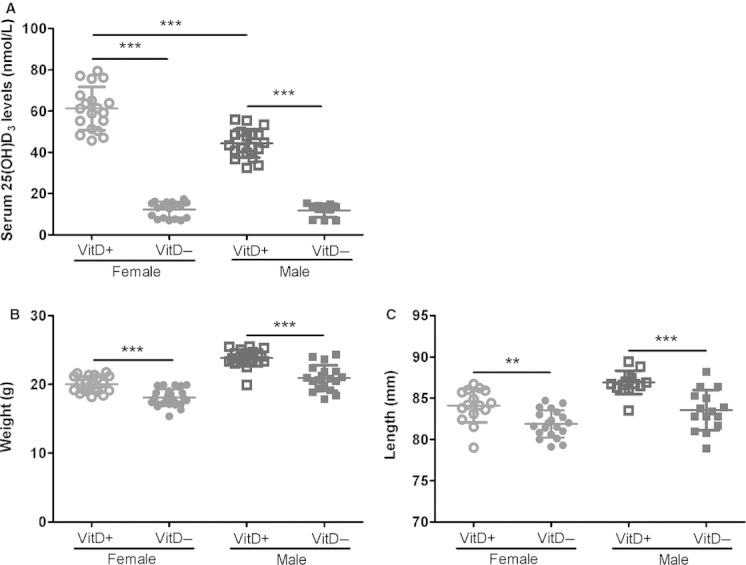
Serum 25(OH)D_3_ levels (A), body weights (B), and snout‐vent lengths (C) in 8‐week‐old vitamin D‐deficient mice and ‐replete mice. Vitamin D‐deficient mice are represented by solid symbols and ‐replete mice by open symbols (females, circles; males, squares). ***indicates *P *<**0.001; **indicates *P *<**0.01. Data are represented as mean (SD), *n* = 12–19/group for serum 25(OH)D_3_ levels; *n* = 20–27/group for body weights and *n* = 13–19/group for snout‐vent lengths.

Mice fed the vitamin D‐deficient diet had significantly lower levels of 25(OH)D_3_ compared to mice fed the ‐replete diet (*P *<**0.001). There was no difference in vitamin D status between vitamin D‐deficient mice of both sexes (*P *=**0.77); however, vitamin D‐replete females had significantly higher levels of serum 25(OH)D_3_ compared with ‐replete males (*P < *0.001; Fig. 1A). 8‐week‐old vitamin D‐deficient mice of both sexes had significantly lower body weights (*P *<**0.001) and snout‐vent lengths (females [*P *<**0.01]; males [*P *<**0.001]) (Fig. 1B and C) compared with their ‐replete counterparts. Serum 25(OH)D_3_ levels were significantly lower in the vitamin D‐deficient dams compared with ‐replete dams (*P *=**0.024; data not shown).

### Lung volume

**Figure 2.**
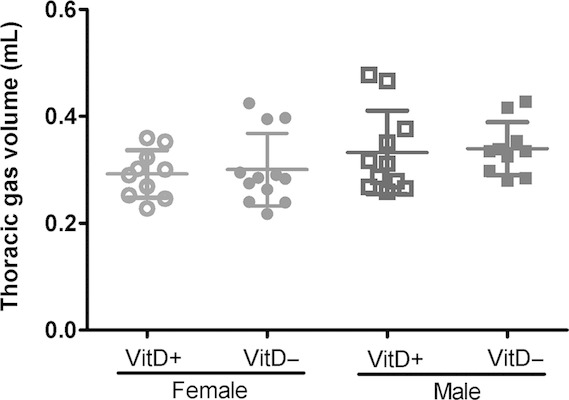
Thoracic gas volumes (TGV) of vitamin D‐deficient and ‐replete mice. Vitamin D‐deficient mice are represented by solid symbols and ‐replete mice by open symbols (females, circles; males, squares). Data are represented as mean (SD), *n* = 10–12/group.

There were no differences in TGV between groups (Fig. 2).

### Methacholine challenge

There were no significant differences in baseline measurements of *R*_aw_ and *H* between vitamin D‐deficient and ‐replete mice of either gender. Baseline *G* was increased in vitamin D‐deficient female mice compared with ‐replete female mice.

**Figure 3.**
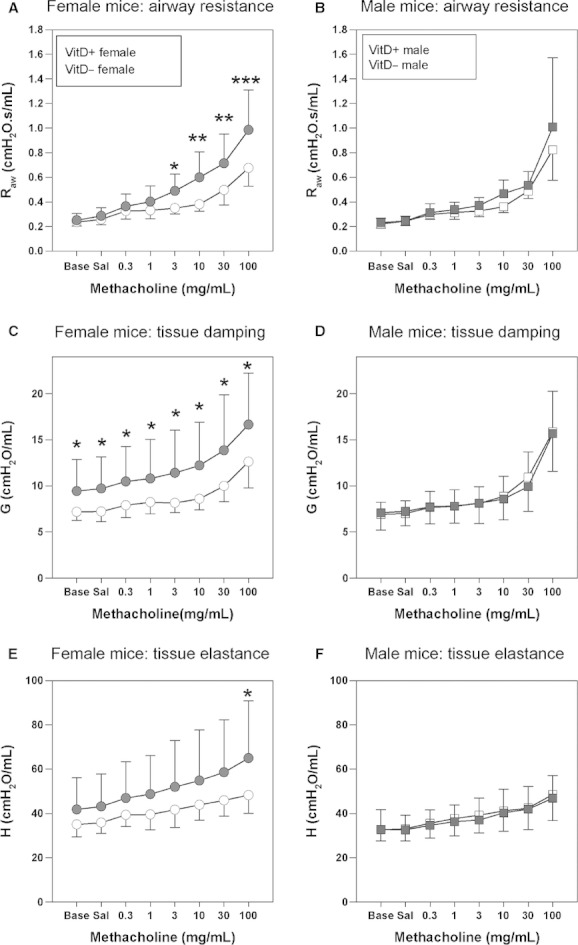
Airway resistance (*R*_aw_; A, B), tissue damping (*G*; C, D) and tissue elastance (*H*; E, F) in response to increasing doses of methacholine in vitamin D‐deficient (female, solid circles; male, solid squares) and ‐replete mice (females, open circles; males, open squares). ***indicates *P *<**0.001; **indicates *P *<**0.01; *indicates *P *<**0.05. Data are represented as mean (SD) *n* = 10–11/group.

An increased response in airway resistance (*R*_aw_) was detected in vitamin D‐deficient females compared to ‐replete females at doses of 3 mg/mL (*P *=**0.04), 10 mg/mL (*P *=**0.001), 30 mg/mL (*P *=**0.003) and 100 mg/mL of methacholine (*P *<**0.001; Fig. 3A). There was no difference in *R*_aw_ between vitamin D‐deficient and ‐replete males (Fig. 3B).

There were no significant differences in tissue mechanics (*G* and *H*) in response to methacholine between vitamin D‐deficient and vitamin D‐replete male mice (Fig. 3D and F). Vitamin D‐deficient female mice had increased *G* compared with vitamin D‐replete females (*P *=**0.048) which appeared to be due to differences at baseline (Fig. 3C). Vitamin D‐deficient females also had increased *H* at 100 mg/mL of methacholine (Fig. 3E).

**Figure 4.**
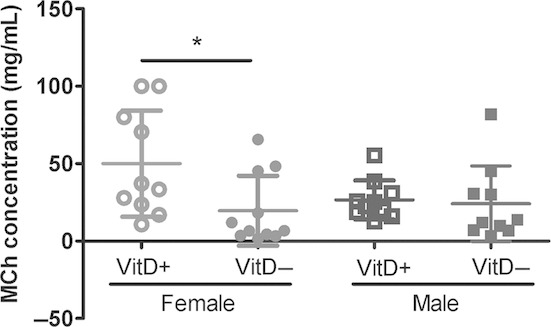
Concentration of methacholine required to elicit a doubling (EC_200_) in airway resistance (*R*_aw_). These values were calculated from the dose–response curves by interpolation (see Fig. 3). *indicates *P *<**0.05. Data are represented as mean (SD), *n* = 10–11/group.

The concentration of methacholine required to elicit a doubling (EC_200_) in *R*_aw_ was significantly lower in vitamin D‐deficient female mice, suggesting an increased sensitivity to methacholine (*P *=**0.02; Fig. 4). No differences were observed in male mice.

### Lung structure

**Figure 5.**
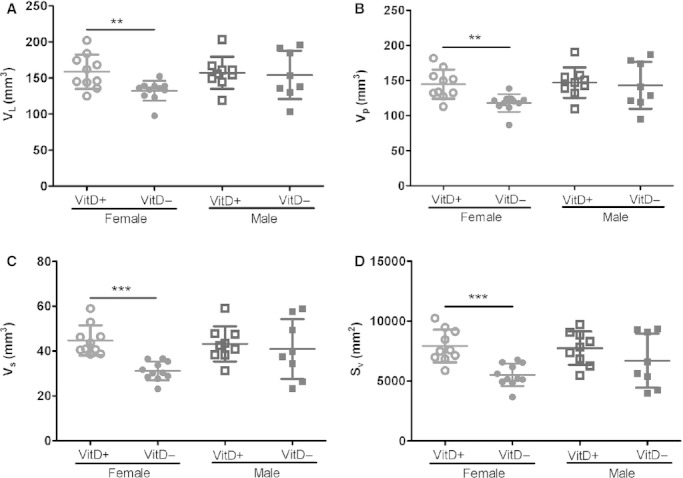
Lung volume (V_L_; A), volume of parenchyma (*V*_p_; B), volume of alveolar septa (*V*_s_; C), and surface area density of alveoli (*S*_v_; D) measured by stereology from fixed lungs of vitamin D‐deficient and ‐replete 8‐week‐old female and male mice. Vitamin D‐deficient mice are represented by solid symbols and ‐replete mice by open symbols (females, circles; males, squares). ***indicates *P *<**0.001; **indicates *P *<**0.01. Data are represented as mean (SD), *n* = 8–11/group.

Post‐fixation and embedding lung volume was significantly smaller in vitamin D‐deficient females compared with ‐replete females (*P *=**0.005; Fig. 5A). The volume of the parenchyma (*V*_p_; Fig. 5B; *P *=**0.002), volume of alveolar septa tissue (*V*_s_; Fig. 5C; *P *<**0.001), alveolar surface area (Fig. 5D; *P *=**0.001), and volume of air in the parenchyma (*V*_a_; *P *=**0.03; data not shown) were also significantly reduced in vitamin D‐deficient female mice compared to ‐replete females. No differences were observed between vitamin D‐deficient and ‐replete male mice.

### ASM mass

**Figure 6.**
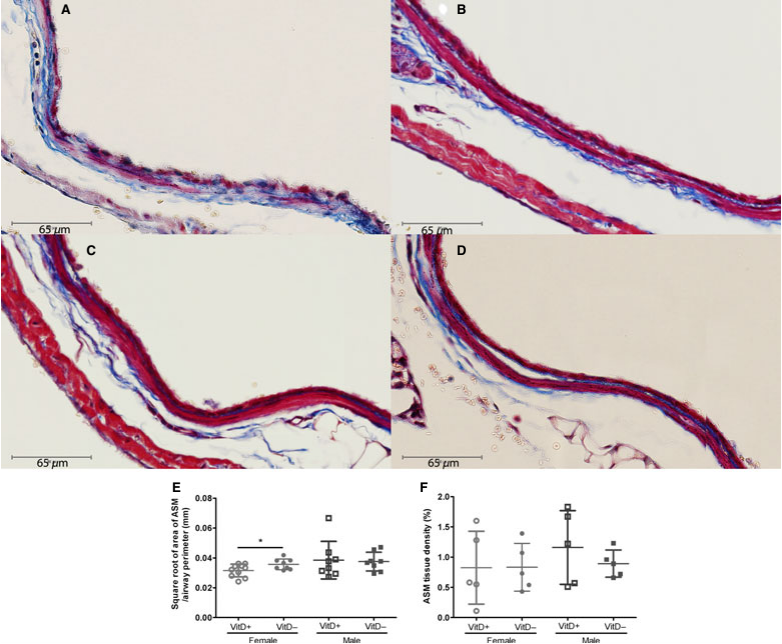
Representation images for airway sections stained with Masson's Trichrome in 8‐week‐old vitamin D‐replete female mice (A), vitamin D‐deficient female mice (B), vitamin D‐replete male mice (C) and vitamin D‐deficient male mice (D). Airway smooth muscle (ASM) mass in 8‐week old vitamin D‐deficient and ‐replete female and male mice (E) and ASM tissue density in embryonic day (E) 17.5 vitamin D‐deficient and ‐replete female and male mice (F) were then quantified. Vitamin D‐deficient mice are represented by solid symbols and ‐replete mice by open symbols (females, circles; males, squares). *indicates *P *<**0.05. Data are represented as mean (SD), *n* = 8–10/group for 8‐week‐old mice and *n* = 5/group for E17.5 mice.

Eight‐week‐old vitamin D‐deficient female mice had significantly higher levels of ASM in the large proximal airways compared with ‐replete female (*P *=**0.04; Fig. 6E). There was no difference in ASM mass in the small distal airways between the groups (data not shown).

Likewise, the tissue density of ASM was not altered by vitamin D deficiency in either female (*P *=**0.98) or male (*P *=**0.38) E17.5 pups (Fig. 6F).

### TGF‐*β* levels

**Figure 7.**
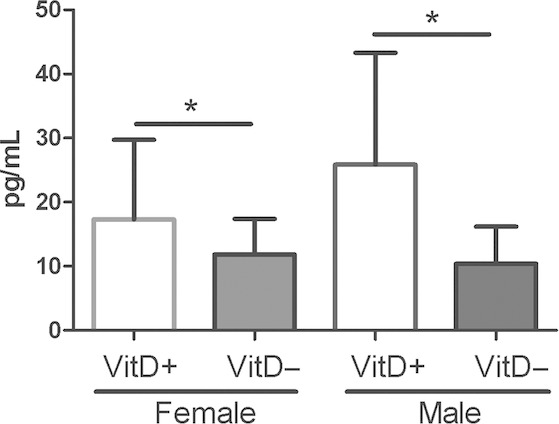
Transforming growth factor *β* levels in bronchoalveolar fluid of 8‐week‐old vitamin D‐deficient and ‐replete male and female mice. Vitamin D‐deficient mice are represented by solid bars and ‐replete mice by open bars. *indicates *P *<**0.05. Data are represented as mean (SD), *n* = 12–13/group.

**Figure 8.**
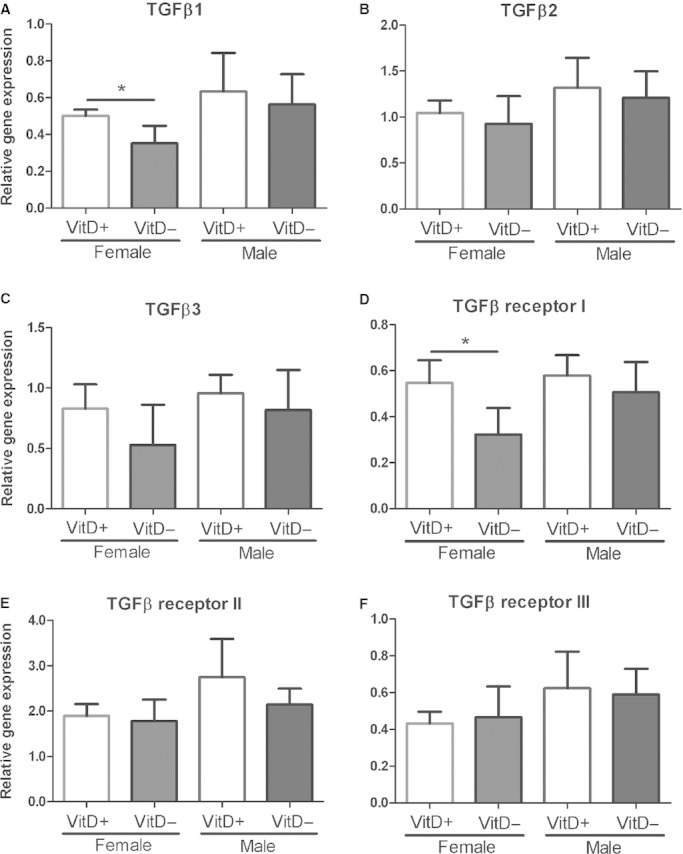
Relative gene expression of transforming growth factor (TGF)*β*1 (A), TGF*β*2 (B), TGF*β*3 (C), TGF*β* receptor I (D), TGF*β* receptor II (E), and TGF*β* receptor III (F) in lungs of embryonic day 17.5 vitamin D‐deficient and ‐replete fetal mice. Data are expressed as fold changes obtained by normalizing candidate genes to the 18s ribosomal RNA reference gene. *indicates *P *<**0.05. Data are represented as mean (SD), *n* = 5/group.

Transforming growth factor‐*β* protein levels were significantly reduced in the BALF of both vitamin D‐deficient male and female mice (*P *=**0.003; Fig. 7). The expression of TGF‐*β*1 (Fig. 8A, *P* = 0.01) and TGF‐*β* receptor I (Fig. 8D, *P* = 0.01) genes were significantly lower in the lung tissue of female vitamin D‐deficient E17.5 mice compared to female ‐replete mice. There were no significant differences in expression of the other genes of the TGF‐*β* pathway in female mice (TGF‐*β*2, *P *=**0.45; TGF‐*β*3, *P *=**0.12; TGF‐*β* receptor II, *P *=**0.64; TGF‐*β* receptor III, *P *=**0.69) at this time‐point. No differences in TGF‐*β* pathway gene expression were detected between male vitamin D‐deficient and ‐replete mice at E17.5 (TGF‐*β*1, *P *=**0.57; TGF‐*β*2, *P *=**0.59; TGF‐*β*3, *P *=**0.42; TGF‐*β* receptor I, *P *=**0.33; TGF‐*β* receptor II, *P *=**0.18; TGF‐*β* receptor III, *P *=**0.76; Fig. 8).

## Discussion

Vitamin D deficiency caused AHR in adult female BALB/c mice. This was accompanied by an increase in ASM mass as well as smaller lung volume and altered lung structure. Male mice appeared to be resistant to these vitamin D‐induced changes in airway structure and responsiveness. A previous study has similarly shown a positive correlation between ASM mass and *R*_aw_ to methacholine in female mice (Plant et al. 2012). Increased ASM has an important role in the pathophysiology of chronic asthma where it has been shown that the thickness of the ASM layer in the airways is related to the clinical severity of asthma and independent of age of onset and duration of asthma (James et al. 2009). Our findings provide clear causal evidence that low circulating vitamin D levels modulate airway remodeling in vivo. This is important as vitamin D deficiency is associated with worse outcomes in asthma (Chinellato et al. 2011; Gupta et al. 2011) and COPD (Janssens et al. 2010), both of which are characterized by airway remodeling (Jeffery 2001). The majority of studies that have attempted to link vitamin D deficiency with outcomes in chronic lung disease have primarily focused on the immunomodulatory effects of vitamin D (Hart et al. 2011). Our study suggests that vitamin D deficiency can directly alter airway structure, which in turn may lead to heightened respiratory symptoms in chronic lung disease. Given that the mice in our study were assessed in the naïve state, this implies that vitamin D deficiency has the capacity to adversely alter disease outcomes independently of its potential impact on immune function.

We have previously shown altered lung structure in 2‐week‐old mice (Zosky et al. 2011). The current study shows that adult 8‐week‐old vitamin D‐deficient female mice have reduced lung volume, reduced volume of parenchyma, alveolar septa and air in parenchymal tissue, as well as reduced alveolar surface area. This indicates that the impairment in lung development at 2 weeks persists into adulthood in females. Mechanistic evidence for a role for vitamin D in lung development has been provided by animal studies where vitamin D induces the maturation of type II alveolar epithelial cells (AECs) and the synthesis of pulmonary surfactant, both of which are key events in fetal lung development. Pulmonary surfactant is important for reducing surface tension and maintaining alveolar integrity (Marin et al. 1993) while the maturation of type II AECs and surfactant synthesis is, in turn, dependent on alveolar epithelial–mesenchymal interactions. Vitamin D influences these interactions by increasing the expression of key homeostatic epithelial–mesenchymal differentiation markers, increasing lipofibroblast and type II AEC proliferation, and decreasing apoptosis (Sakurai et al. 2009). TGF‐*β* signaling is also implicated in epithelial–mesenchymal interactions (Ramirez et al. 2010), and is regulated during lung development and alveolarization (Warburton et al. 2013). As vitamin D can modulate TGF‐*β* signaling (Ramirez et al. 2010; Luderer et al. 2013), and TGF‐*β* is associated with ASM proliferation (Chen and Khalil 2006), we investigated if TGF‐*β* levels were altered in our mouse model of vitamin D deficiency.

TGF‐*β*1 protein levels in the BALF of vitamin D‐deficient male and female mice were lower compared with vitamin D‐replete mice. Impaired activation of TGF‐*β* signaling has previously been shown in a mouse model of vitamin D deficiency (Luderer et al. 2013) and there are in vitro studies demonstrating that 1,25(OH)_2_D_3_ upregulates the expression of TGF‐*β*1. TGF‐*β*1 concentrations were increased in the BALF of asthmatics compared with nonasthmatic controls (Redington et al. 1997). TGF‐*β*1 is a known mediator of airway remodeling and increases the proliferation of ASM (Chen and Khalil 2006). Thus, our data appear to be contrary to these observations. However, the effects of TGF‐*β*1 on cell proliferation are complex and dependent on the local environment. For example, TGF‐*β*1 inhibits ASM cell proliferation in subconfluent culture, but promotes proliferation when the cells are confluent (Okona‐Mensah et al. 1998). Furthermore, TGF‐*β* induces ASM cell proliferation at low doses but inhibits proliferation at higher doses (Battegay et al. 1990; Halwani et al. 2010). It is, therefore, possible that reduced TGF‐*β*1 expression during lung development may contribute to the increased ASM mass due to the complexities of the in vivo environment. Alternatively, the decrease in TGF‐*β* may be a homeostatic response as the lung attempts to rectify the increase in ASM in response to vitamin D deficiency.

Although the lower levels of TGF‐*β*1 in the BALF of vitamin D‐deficient mice were observed in both males and females in our study, lung structure and function differences were only observed in female mice. Clearly, there are distinct sex‐related differences in the structural and physiological responses to vitamin D deficiency. Cross‐talk between vitamin D and TGF‐*β* signaling (Subramaniam et al. 2001) as well as a functional synergy between the vitamin D and estrogen endocrine systems (Correale et al. 2010), may explain why differences were only observed in females. Furthermore, as observed previously (Gorman et al. 2012a), vitamin D‐replete male mice did not have the same levels of serum 25(OH)D_3_ compared with ‐replete females in the current study. There are several possible explanations for this observation. Male mice may have a reduced capacity to produce or retain 25(OH)D_3_, or have an increased rate of breakdown of 1,25(OH)_2_D_3_ into other less active metabolites. This is supported by evidence that the expression of 1,25(OH)_2_D_3_ synthesis and its breakdown enzymes, (CYP27B1 and CYP24A1, respectively) are upregulated in male vitamin D‐deficient mice, indicating a faster rate of 1,25(OH)_2_D_3_ metabolism and catabolism (Gorman et al. 2012a). Importantly, our data are consistent with previous studies investigating the effects of gender on AHR in mice which have shown that the increases in *R*_aw_, *G,* and *H* in response to methacholine are generally greater in naïve male mice than naïve female mice (Card et al. 2006). One intriguing possibility is that the lower levels of vitamin D, in male mice, may result in increased ASM, and a greater naïve response to broncho‐constricting agents.

Reduced somatic growth was observed in vitamin D‐deficient mice of both genders. In health, body size is well‐known to be strongly associated with lung volume (Cook et al. 1958), however, we found no difference in in vivo lung volume in vitamin D‐deficient male mice despite their lower body lengths and weights, clearly highlighting the importance of direct measures of lung volume when comparing the effects of an intervention that may impact on somatic growth. Interestingly, while there was no difference in TGV measurements, lung volume measured stereologically was smaller in vitamin D‐deficient female mice. It is important to recognize that TGV measurements in vivo are made at EELV, whereby the total volume of lung in the air is a result of the opposing forces generated by the inward elastic recoil of the lung and the outward force generated by the chest wall (Zosky et al. 2011). This discrepancy between in vivo and ex vivo measurements of lung volume in the females suggests that either 1) the elastic recoil of the lung parenchyma was higher in vitamin D deficiency such that the lung shrunk to a greater extent after being removed from the chest, or 2) there was gas trapping at EELV in the female vitamin D‐deficient mice resulting in a higher TGV in vivo. While we are not able to distinguish between these possibilities, it is clear that there is an impact of vitamin D deficiency on airway responses to methacholine despite the apparent lack of an effect of deficiency on lung volume in vivo; at least in the female mice.

Our findings of altered lung structure and function in adult female mice led us to further investigate whether this was a result of developmental deficits due to vitamin D deficiency in utero. While we did not find a difference in ASM density between vitamin D‐deficient and ‐replete mice, we found that the relative gene expression of TGF*β*1 and TGF*β* receptor I was downregulated in vitamin D‐deficient female E17.5 pups. It is possible that any effect on ASM mass due to vitamin D deficiency may not have been detectable at E17.5 as maturation of ASM may continue through to adulthood despite ASM being laid down in the fetal mouse lung at E12 (Tollet et al. 2001). TGF‐*β*1 is important for lung branching in the fetal mouse and mediates epithelial–mesenchymal interactions in the developing lung by colocalization with extracellular matrix proteins (Bartram and Speer 2004). Reduced expression of TGF‐*β*1 in the developing mouse lung as a result of vitamin D deficiency may affect lung branching morphogenesis and lead to abnormal development. This hypothesis is consistent with the altered expression of this pathway in the females at E17.5 and increased AHR and ASM at 8 weeks of age; an association that was absent in the male mice.

In summary, vitamin D deficiency contributes to AHR and airway remodeling in adult female mice. Specifically, vitamin D deficiency caused an increase in ASM mass which was in turn associated with an increased response to methacholine. While this effect was not observed in males, it was interesting to note that the overall levels of vitamin D in the male ‐replete mice were lower than the female ‐replete mice which also corresponded to higher levels of ASM in the males and a greater response to methacholine. Vitamin D deficiency also caused a reduction in TGF‐*β*1 protein levels in both male and female mice, as well as reduced gene expression of TGF‐*β*1 and TGF‐*β* receptor I in female E17.5 fetal pups. These observations may provide a mechanism by which vitamin D deficiency to chronic lung disease that is unrelated to the immunomodulatory effects of vitamin D.

## Acknowledgments

We would like to acknowledge T. Iosifidis for conducting the *α*‐smooth muscle actin immunohistochemistry.

## Conflict of Interest

None declared.
